# Dynamical Role of Pivotal Brain Regions in Parkinson Symptomatology Uncovered with Deep Learning

**DOI:** 10.3390/brainsci10020073

**Published:** 2020-01-30

**Authors:** Alex A. Nguyen, Pedro D. Maia, Xiao Gao, Pablo F. Damasceno, Ashish Raj

**Affiliations:** 1 Department of Radiology and Biomedical Imaging, UC San Francisco, San Francisco, CA 94107, USA; alex.nguyen@ucsf.edu (A.A.N.); xiao.gao@ucsf.edu (X.G.); pablo.damasceno@ucsf.edu (P.F.D.); 2 Bakar Computational Health Sciences Institute, UC San Francisco, San Francisco, CA 94158, USA; 3 Center for Intelligent Imaging, UC San Francisco, San Francisco, CA 94107, USA

**Keywords:** Parkinson, deep learning

## Abstract

*Background:* The release of a broad, longitudinal anatomical dataset by the Parkinson’s Progression Markers Initiative promoted a surge of machine-learning studies aimed at predicting disease onset and progression. However, the excessive number of features used in these models often conceals their relationship to the Parkinsonian symptomatology. *Objectives:* The aim of this study is two-fold: (i) to predict future motor and cognitive impairments up to four years from brain features acquired at baseline; and (ii) to interpret the role of pivotal brain regions responsible for different symptoms from a neurological viewpoint. *Methods:* We test several deep-learning neural network configurations, and report our best results obtained with an autoencoder deep-learning model, run on a 5-fold cross-validation set. *Comparison with Existing Methods:* Our approach improves upon results from standard regression and others. It also includes neuroimaging biomarkers as features. *Results:* The relative contributions of pivotal brain regions to each impairment change over time, suggesting a dynamical reordering of culprits as the disease progresses. Specifically, the Putamen is initially the most critical region accounting for the overall cognitive state, only being surpassed by the Substantia Nigra in later years. The Pallidum is the first region to influence motor scores, followed by the parahippocampal and ambient gyri, and the anterior orbital gyrus. *Conclusions:* While the causal link between regional brain atrophy and Parkinson symptomatology is poorly understood, our methods demonstrate that the contributions of pivotal regions to cognitive and motor impairments are more dynamical than generally appreciated.

## 1. Introduction

Machine Learning (ML) is quickly affirming itself as a valuable tool for Parkinson’s Disease (PD) diagnosis [1,2,3,4]. ML methods typically learn specific sets of functions to perform data categorization, with deep-learning networks being able to detect more complex patterns in massive datasets [5,6]. Numerous studies have leveraged the predictive capabilities of deep-learning models for Alzheimer’s symptomatology [7,8,9,10]. However, the high variability of Parkinsonian brains, the smaller sample size compared to Alzheimer’s datasets, and the lack of longitudinal atrophy progression for individual patients pose a challenge even to state-of-the-art machine-learning methods. 

The first ML studies with PD patients were aimed at predicting their current condition or cognitive/motor scores using baseline parameters such as demographic data, CSF, and blood protein analysis, speech, movement, or genetics [11,12,13]. Here, we estimate *future* motor and cognitive states of PD patients using an autoencoder deep-learning model to reduce the dimensionality of the problem [5]. While other ML studies in PD also report high levels of accuracy [14,15,16], our focus is to highlight the brain regions that play a pivotal role in the model-based predictions. We show that their relative contribution to the observed symptoms changes over time, suggesting a dynamical reordering of culprits for each functional impairment as the disease progresses. This provides new insight to the poorly understood causal link between regional atrophy and PD symptomatology. The high accuracy levels reported herein indicate that ML models such as ours, once trained on a larger dataset than currently available publicly, could also be effective as a prognostic clinical tool.

**Highlights**

- We implemented an autoencoder neural network for the prediction of future motor/cognitive impairment
- Our best model prediction performed with 85% accuracy, 80% specificity, and 100% sensitivity
- The autoencoder significantly improves upon standard statistical regression results
- Pivotal impaired regions change their relative contributions to Parkinsonian symptomatology over time

## 2. Material and Methods

***PPMI Study Data:*** All data used in this study was obtained from the Parkinson’s Progression Markers Initiative (PPMI) database [17]. We selected 42 healthy controls (27 males, 15 females, age = 59.3 ± 11.0, and MoCA = 28.5 ± 1.2) and 116 PD patients (74 males, 42 females, age = 59.8 ± 9.5, MoCA = 27.5 ± 2.1, MDS-UPDRS-III = 20.6 ± 8.6) that had complete baseline features. Within the initial 116 PD patient cohort, the mean duration of disease at the time of baseline is 6.26 months (±6.25). The fraction of patients for left/right/symmetric side dominance is 24/86/6. Scores for MDS-UPDRS-III were acquired when patients were off medication [18]. Subjects missing more than two input features were not included in our analysis.

***Structural MRI acquisition:*** Structural MRI data acquisition occurs at baseline and is used to predict future outcomes in PD patients. PPMI protocol requires a 3-dimensional T1-weighted scan, using either a Magnetization Prepared Rapid Gradient Echo (MPRAGE) or a Spoiled Gradient Recalled (SPGR) sequence [17]. The field of view must include the vertex, cerebellum, and pons, and have a slice thickness of no greater than 1.5 mm without an interslice gap. The repetition and echo times are set according to the individual manufacturer’s recommendations. Sagittal 3D T1-weighted MR images were obtained with the following parameters: slice thickness 1.2 mm, slice gap 0 mm, voxel size 1 mm × 1 mm × 1.2 mm, and matrix 256 × 256 × 170–200. Data were acquired at PPMI centers using scanners from three different manufacturers (GE, Chicago, IL, USA; Siemens, Erlangen, Germany and Philips, Amsterdam, The Netherlands). Additional details regarding these subjects, including study inclusion and exclusion criteria, are available at the PPMI website (www.ppmi-info.org/data).

***DBM and Brain Connectome:*** The main features used in our model are deformation-based morphometry (DBM) values, which consists of a calculation of regional brain atrophy based on the displacement to a standard MNI152-2009c parcellation template provided by Zeighami et al. [19]. DBM, also referred to as the determinant of the Jacobian transformation matrix, was used to calculate the local change in tissue density. This method spatially transforms the MRI data to a stereotaxic template linearly and then non-linearly [19]. This change in local deformation is measured and presented as atrophy maps. The baseline DBM atrophy map includes 78 brain regions, since atrophy values from cerebellum regions are not considered. While PPMI also acquired Diffusion-weighted imaging (DWI) images, which are arguably more sensitive compared to structural imaging [3,20], we opted for using MRI-derived atrophy dataset to facilitate comparisons with previous studies and to ensure that our results were not driven by our use of a different imaging modality.

**Figure 1:**
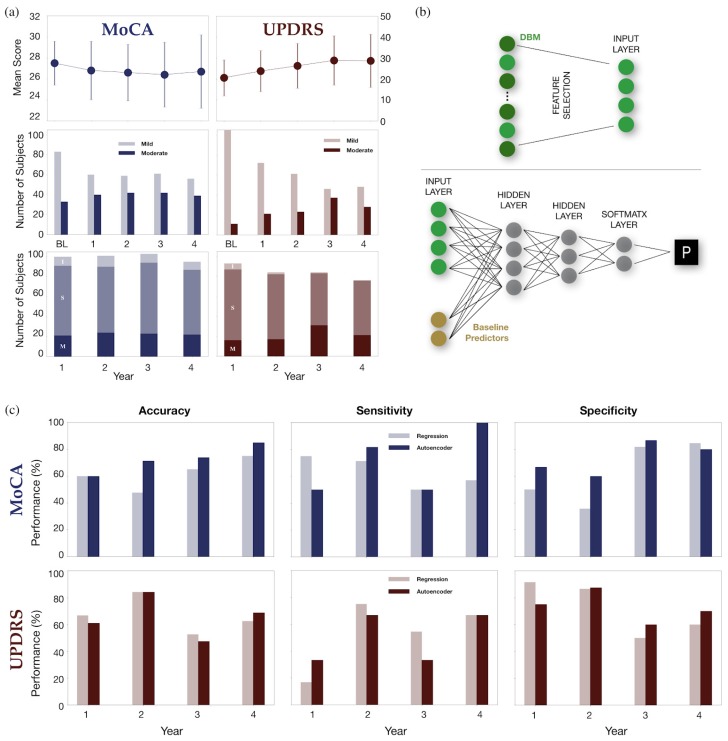
(**a**) Average Montreal Cognitive Assessment (MoCA) scores over time (top left), number of subjects in each category at baseline (BL) and following years (mid left), and observed change in cognition over the years (bottom left). I, S, M mean Improved, Stable, and Moderate Impairment, respectively. Analogous plots for MDS-UPDRS-III scores on the right. (**b**) Architecture of the implemented two-step deep autoencoder neural network (NN). Deformation-based Morphometry (DBM) features were selected using a Spearman’s correlation statistical test (top) and then used as input, along baseline predictors, for the NN (bottom). The two hidden layers were comprised of stacked sparse autoencoders using the logistic sigmoid activation function. A final SoftMax layer was used for classification. (**c**) Performance of MoCA (top, blue) and UPDRS (bottom, red) predictions for test sets in both regression (light tone) and Autoencoder (dark tone) models. Accuracy, sensitivity, and specificity are shown from left to right.

***Predicted outcome variables:*** The predicted Montreal Cognitive Assessment (MoCA) scores were classified into two categories: normal cognition vs. mild cognitive impairment. This choice was based on the work of Hoops et al. [21], where the cutoff score (26/27) effectively differentiated the two groups. The predicted MDS-UPDRS-III scores were classified into two categories as well: mild motor impairment vs. moderate motor impairment, using the optimal cutoff score (32/33) validated by Martínez-Martín et al. [22]. All figures and text with UPDRS refer solely to MDS-UPDRS-III. These categorical outcomes are used over the individual raw scores due to the wide variability of raw scores as well as disease progression. Figure 1a shows the averaged MDS-UPDRS-III and MoCA scores over four years.

***Logistic regression and Autoencoder:*** Logistic Regression (LR) is a powerful statistical algorithm for binary categorization and a natural choice for classifying moderate vs. mild symptomatology in PD patients. It will perform poorly, however, if the classification requires non-trivial combinations of the input features. In this scenario, neural networks may excel if enough training samples are available. Thus, we opted to implement a LR model and investigate whether the classification could be improved by employing a neural network architecture to the same dataset.

***Strategies to avoid overfitting:*** The number of parameters in neural network models scales with the number of input features and with the number of neurons used in each layer. Since the number of input features in our model is of the order of the number of cases in our training set, we combine two strategies to avoid overfitting: feature selection and an autoencoder model. To select features, we calculated the Spearman’s rank correlation coefficient between the DBM features and the motor/cognitive scores, removing those with p values above 0.05 (see Table 1 and Table 2). The selected features are then combined with baseline demographics and biospecimen measurements to create the input layer that is then fed into a deep autoencoder network.

***Network architecture:*** Autoencoders are compression algorithms that learn a reduced representation of the input data by minimizing the loss of information when decoding the compressed data [23]. We analyzed the information loss in the input vector as a function of the data compressibility (adjusted mean square error function with L2 and sparsity regularizers) and empirically select a Pareto optimal compression. In our case, this compressed the data into a new vector containing only 20 and 10 features, respectively, to be fed into two hidden layers with 20 and 10 neurons each. Each layer was followed by a logistic sigmoid function, which empirically outperformed both simple and positive saturating linear transfer functions. See Figure 1b for details.

Our fully connected autoencoder uses backpropagation and scaled conjugate gradient descent for training. The global error cost function of the proposed autoencoder is based on the adjusted mean square error function. First, the 78 brain atrophy regions and the 78 predicted atrophy rates are reduced to a smaller representation of data by only selecting statistically significant features based on Spearman’s rank correlation coefficient. The reduced imaging features with and without the addition of the analytical projections obtained from the NDM are combined with baseline demographics and biospecimen measurements. Stacking of the individual sparse autoencoders at each layer creates a deep autoencoder network. A supervised SoftMax classification layer is used to link the input to the desired output. The SoftMax layer generates a probability matrix for each possible class. The entire deep autoencoder model is finely tuned at the end so that it only takes one iteration to improve all the weights simultaneously. To fine tune the model, we implement backpropagation on the entire stacked autoencoder network by retraining it on the training data in a supervised fashion. The starting weights and biases are set randomly at the start of each model training with a normal distribution of a small value close to, but not equal to zero.

***Initialization and adjustment of hyperparameters:*** Network weights were randomly initialized from a Gaussian distribution with zero mean and small variance and optimized via scaled conjugate gradient descent [24]. Optimal hyperparameters were adjusted in a sequential search by changing one variable at a time [25]. Performance results from the training and validation sets were recorded (see Figure 1c) and the best hyperparameters saved for the final performance evaluation on the test set.

To prevent overfitting of the training data, we declare specific parameters for the L2 weight regularization, sparsity regularization, and sparsity proportion coefficients terms onto the cost function at the start of model training. The range of values tested for L2 weight regularization, sparsity regularization, and sparsity proportion are 0.001 to 0.009, 2 to 6, and 0.05 to 0.20, respectively. L2 regularization helps drive outlier weights closer to zero. By regularizing or shrinking the L2 weight coefficient, prediction accuracy may be improved, and the variance may be reduced. The larger the sparsity regularization parameter, the greater its impact on activated training data. The sparsity proportion specifies the amount of training examples a neuron reacts with, whereas the sparsity regularization term controls the impact of sparsity for faster optimization and evaluation of the model. By implementing this restriction on sparsity, this pressures the neural network to reduce and store only the essential features of the data.

***Testing and cross-validation:*** For out-of-sample testing, we used 5-fold cross-validation: we randomly split the data 5 different ways between a training (80%) and a hold-out testing set (20%) for prediction purposes. All the data extraction, neural network modeling, and statistical analysis were performed with MATLAB R2018a (MATLAB, Natick, MA) on an Intel Core i7-2620M CPU @2.7GHz and 8GB RAM.

***Linking regional brain atrophy to Parkinsonian symptomatology:*** To evaluate how strongly brain region **j** affects motor/cognitive classification at a given year, we increase this region’s DBM value (either by 10% or 20%) and record the corresponding impact on the prediction matrix P:

δ(j) = || P_original_ – P _w/DBM increase in region **j**_**||**, (1)

where || || denotes the Frobenius matrix norm. We then use the normalized vector δ to rank the most-impacting regions for each modality over time.

## 3. Results

Figure 1c shows the prediction accuracy, sensitivity, and specificity for classifying patients into two cognitive categories (normal vs. mild impairment) based on their MoCA scores. Light tone colors correspond to LR and darker tones to the autoencoder model. Their efficiency varies across all future time points, with worse results at the first years and best ones at the last year. The autoencoder method has the potential to correctly predict future cognitive categories with up to 80% accuracy, significantly improving LR predictions. Figure 1c also shows the prediction accuracy for classifying the MDS-UPDRS-III motor scores into two categories (mild vs. moderate decline) over the years. For this case, the two methods performed similarly on all categories.

**Figure 2:**
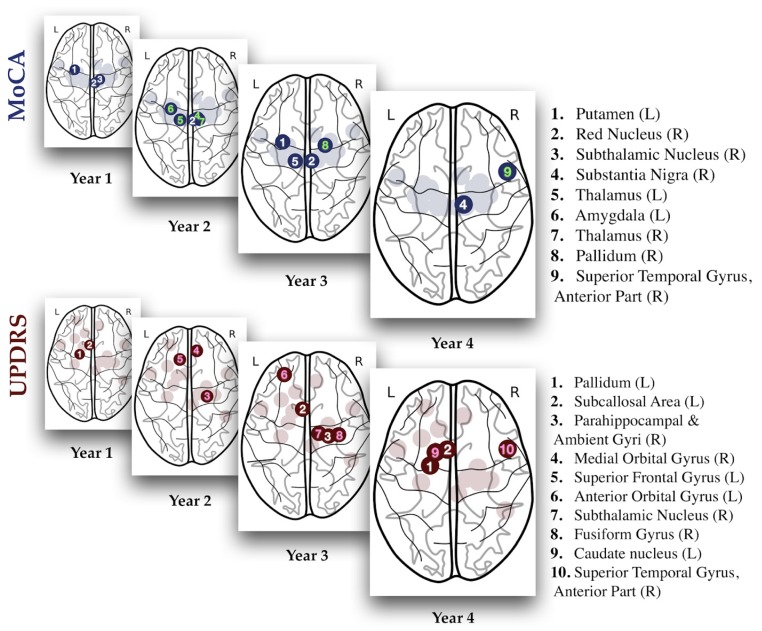
Emergence of distinct salient regions when predicting MoCA (top) and UPDRS (bottom) scores for different years. Numbered regions represent areas with relative saliency above a 4% threshold and are highlighted whenever they first appear, compared to the previous years. Shaded background regions correspond to the range of brain regions used as input for the neural network. A list of complete regions and their relative saliencies is shown in Figure 3.

Figure 2 highlights the regions with highest impact (saliency) in the model predictions. The phase space of all regions comprising the input layer is shown as shaded areas in the background. For both MoCA and UPDRS, a brain area is numbered if its contribution to the model prediction, relative to all the other contributing areas, exceeds a threshold (ΔP_j_ > 0.04). We highlight areas as they appear for the first time, relative to previous years. As it can be seen, a different set of regions is involved in predicting patient future classification, with no single region being salient across all four years of classification.

**Figure 3:**
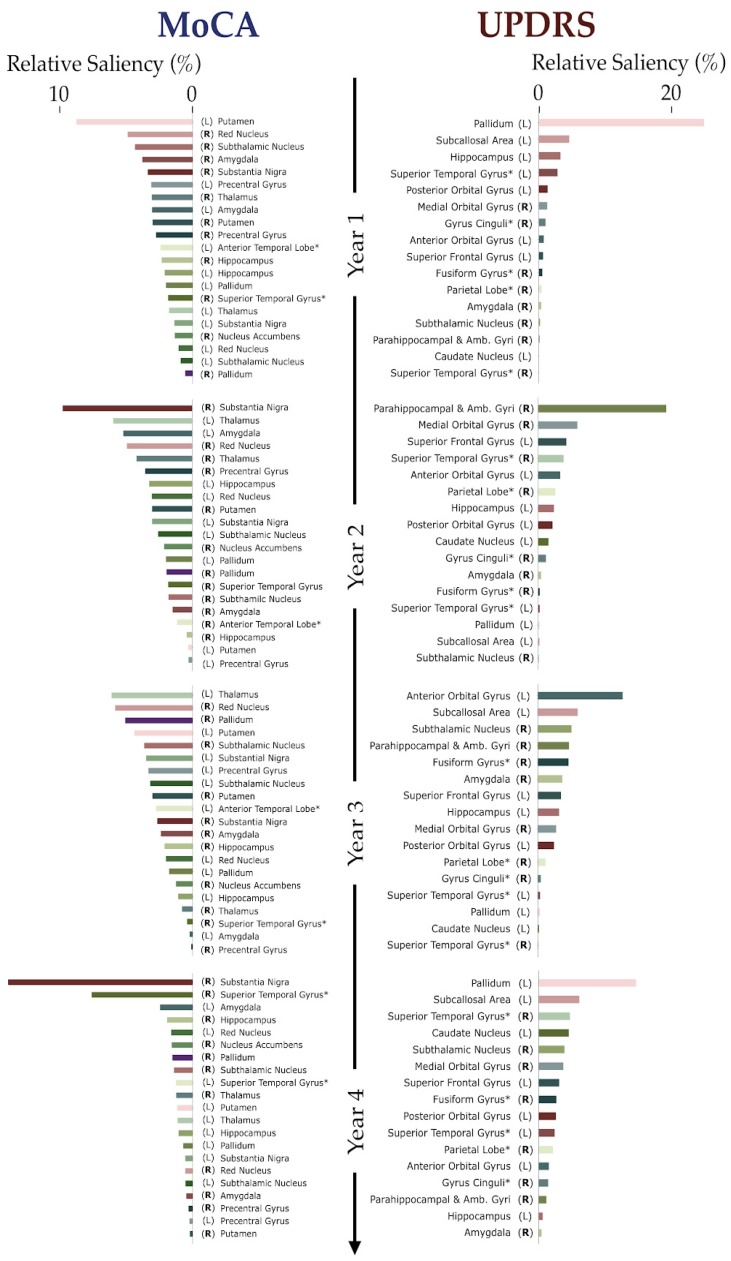
Relative saliency of different regions over time. From top to bottom, percentual value of saliency for each neural network predicting MoCA (left) and UPDRS (right) for all 4 years 1–4. Colors follow each region and show the reordering of principal salient regions responsible for the score predictions. Right hemisphere regions are emphasized to show that similar reordering also happens in the level of the whole brain. See SI for full names of abbreviated regions.

Figure 3 provides a quantitative assessment of the saliency regions for each year, showing all (i.e., non-thresholded) areas from the NN’s input layer. For each model prediction (MoCA and UPDRS), regions are uniquely colored and then ranked by their relative contribution to that year’s prediction. The reordering of colors, observed from year to year, illustrates the malleable nature of this ranking, emphasizing how the roles played by regions significantly differ depending on the stage of PD progression being predicted.

## 4. Discussion

In this study, we predicted future motor and cognitive states of a patient using only features collected at baseline. We implemented and finely tuned an autoencoder deep-learning model to significantly improve the results from standard LR. Our model can predict cognitive and clinical status of de novo PD patients over a period of 1 to 4 years. Our approach relies on well-established neuroimaging software pipelines and is based on low-dimensional features derived from regional DBM. Consequently, the training and testing of our model is far quicker and computationally inexpensive compared to full-stack neural networks that ingest entire imaging volumes and perform prediction on voxel-level information.

### 4.1. Rationale for Variable Selection

Our autoencoder predicts a patient’s cognitive score and motor score over a few years based on anatomical brain features recorded at baseline. We choose the MoCA over the Mini Mental State Examination due to is better sensitivity [21,26], and differentiate three cognitive stages of PD: no cognitive impairment, mild cognitive impairment, and dementia. A few shortcomings of MoCA datasets include the clinicians’ biases at the time of testing and the cumulative scoring basis of its subtests [26]. Undergoing studies are trying to improve the MoCA scale to better reflect the cognitive state of different PD patients. Regarding our motor-score predictions, we use the Unified Parkinson’s Disorder Rating Scale Part III (MDS-UPDRS-III) as it provides an efficient and reliable test to evaluate the motor capabilities of PD subjects [27]. In fact, Greffard et al. [28] demonstrated that this score is linearly linked to neuronal density, which in turn, may reflect neuronal damage and regional atrophy.

In this study, we have eschewed the most commonly used approach in AI, which is the use of voxel-level entire imaging datasets as features. We have instead elected to employ regional volumetric information available from established neuroimaging analysis pipelines, in this case, using the DBM measure of regional atrophy. This choice certainly entails some loss of information but also leads to a tremendous reduction in dimensionality—which we consider is essential when using NN models on limited training samples. In the field of medical imaging, the number of training examples is practically and ethically severely limited, and we believe an evidence-based dimensionality reduction is essential. Indeed, regional volumetric analysis is bread-and-butter in the field of neuroimaging, especially for degenerative disease, which are highly stereotyped spatially. Thus, by exploiting a priori neurophysiological knowledge regarding regional distribution of atrophy in PD, we were able to overcome high dimensionality without losing disease-relevant information.

Among the various available options for regional volumetric analysis, we chose DBM [19]. For this study, we chose to use an existing set of brain connectomes from healthy subjects as previously reported in Zeighami et al. [19]. This choice was dictated by our desire to consider the most high-quality connectomes, which the healthy connectome dataset provides. As we have previously argued [29,30], it is usually sufficient to consider an average template healthy connectome for the purpose of graph modeling, since the disease cohort under investigation is a de novo (i.e., early) group of PD patients, whose connectome architecture may be considered relatively unimpaired. Studies show that DBM is superior to voxel-based morphometry (VBM) in detecting subcortical irregularities in the temporal lobe during epilepsy [31]. Moreover, VBM is less sensitive to subcortical atrophy and may not accurately reflect MRI data, justifying the adoption of DBM features in all models. DBM may also be superior on the current datasets to the common Freesurfer-based regional volume and cortical thickness measures [32], since PPMI subjects are de novo early-stage patients whose brain atrophy has not yet progressed to the point where large effect sizes in thickness or volumes can be sensitively measurable. DBM might also be more appropriate for subcortical structures where Freesurfer typically is less reliable than on the cortex.

### 4.2. Pivotal Regions for Cognitive and Motor Decline Prediction

The upper plots in Figure 2 show influential areas identified by the neural network model as “salient”: essential areas used by the NN model for classifying patients according to their cognitive decline over the years. Those areas are well corroborated by current literature: Melzer et al. [33] reported a grey matter reduction in the Putamen, among other areas, of PD patients with mild cognitive impairment. The Substantia Nigra (pars compacta) along with the nigrostriatal dopaminergic pathways are also likely compromised in PD [34]. Cognitive symptoms, such as intellectual fatigability, decreased verbal fluency, and discrete memory impairment, have been reported in relation to Red Nucleus deficits [35,36]. Finally, increased activity in the Subthalamic Nucleus activity was reported during decisions requiring high cognitive burden and when linking cognitive and motor processes [37]. The last glass brain in Figure 2 for MoCA score predictions shows that four years after baseline, the new most-impacting regions for MoCA are the Substantia Nigra and the Superior Temporal Gyrus. This is again consistent with significant changes in grey matter reported in PD patients with MCI [38,39]. Amygdala was also a salient region in these analyses. Mesial and surrounding temporal cortices, and amygdala, are well known as sites that govern cognitive and memory functions in the brain, hence their involvement as salient regions for the prediction of MoCA is expected.

Figure 2 also shows the key regions responsible for UPDRS classification. During the first year, those are the Pallidum and the Subcallosal Area. The Parkinsonian state is indeed characterized by alterations in the temporal-spatial processing of information within the Pallidum [40]. For fourth year predictions, the Caudate Nucleus become significant, consistent with descriptions of its key involvement in motor responses [41,42,43,44,45,46]. In fact, animal studies show that the Caudate Nucleus contributes significantly to the accuracy of directed movements [47]. Additionally, a large study with elderly patients linked volume reduction of the Caudate Nucleus to age related decline of motor performance [48]. The scoring of UPDRS-III reflects four most classic Parkinsonism syndromes: tremor, rigidity, akinesia, and postural disturbances, which could be well explained by the death of dopaminergic neurons in Substantia Nigra. Our method ranked the parahippocampal and ambient gyri as pivotal regions for UPDRS-III, which is not strongly supported by the literature as these regions are usually considered cognition rather motor related [49]. The inclusion of these regions might be capturing, instead, a more general reflection of PD-related disability.

### 4.3. Dynamical Reordering of Culprits in Parkinsonian Symptomatology

Figure 3 shows that the relative contributions of different brain regions to either MoCA and UPDRS scores change over time, suggesting a dynamical re-accentuation of regional contributions for each symptom. Regarding the MoCA scores, the Red Nucleus (R) plays a consistent role throughout years one to three, but a minor role in year four. The hippocampal contribution to MoCA, on the other hand, seem to increase consistently over the years. There is no clearly dominant salient region until the fourth year when the Substantia Nigra (R) dominates all others. Interestingly, the ML method is also most accurate in this year. In all years, most significant contributions come, primarily, from the right brain hemisphere, although dynamical rearrangement is also observed to occur.

For the UPDRS, we notice a substantial contribution of the Pallidum (years = 1,4) and a dominant contribution of the parahippocampal and ambient gyri in the second year that decays afterward. The role of the subcallosal area also grows over the years. As in the MoCA prediction, the UPDRS prediction is also more accurate when there is a clearly dominant salient region (year = 2). Finally, while the contributions in the first year come mostly from the regions in the left hemisphere, we observe a mixed contribution in subsequent years. There is no apparent relationship between the salient regions and the dominant side of the patient since the number of patients with right/left/symmetric dominance is 68/21/4 in our cohort. These results strongly suggest that a dynamic reordering of salient regions governs an evolving landscape of cognitive and motor dysfunction in PD. This aspect has received almost no attention in the field, but one that we believe may be critical in a fuller understanding of PD progression. A practical benefit from this study may be that a neurologist may become more motivated to probe in more detail the specific patterns of atrophy in these selected salient regions in their patients’ brain. The salience of these regions also raises several unanswered questions about their role in downstream cognitive and clinical disability. Some of these regions may be involved in neurological and cognitive resilience in PD—an aspect that merits further investigation.

### 4.4. Limitations and Future Work

The PPMI database is one of the largest of its kind, but the number of patients with significant longitudinal data is still small, precluding the application of several other machine-learning models that require larger training sets. The validation of the autoencoder model was also restricted to a narrow timespan range (1 to 4 years past baseline). There is a wide variability in atrophy patterns as well as cognitive and motor scores across PD patients. Specifically, there may be significant biases from the clinicians and/or study centers when scoring the MoCA and MDS-UPDRS-III tests. A more comprehensive PD diagnosis and prognosis performed by neuropsychologists could dramatically improve the training of the neural network. It is expected that our model will also improve its predictive power with the increase of the dataset. In fact, new patients had their baseline measurements added to the dataset and their longitudinal data is currently being processed.

Dopaminergic neurons loss in the Substantia Nigra (SN) has been widely considered to be the cause of motor symptoms in PD. This study, however, was not able to detect SN as a salient region for UPDRS classification. One possible explanation is that the neural network may find overall high levels of atrophy across patients redundant for motor-score prediction when other sources of discrepancies between motor versus non-motor PD patients are available. In other words, while the strong SN atrophy is almost certainly contributing to the high mean UPDRS of our cohort of patients, the relative differences in UPDRS are less correlated with differences in SN atrophy than they are to atrophy in the regions shown in Figure 2 and Figure 3.

An interesting avenue for future work is to explore promising imaging biomarkers from white matter, including those related to demyelination (e.g., from T2 weighted MRI), and altered fiber integrity (from diffusion weighted MRI). In fact, Bouhrara and colleagues [50] have shown that the brainstem undergoes demyelination with aging, and have indicated that this may contribute to the structural changes observed with aging. Since myelin loss is involved in PD, this modality could be applied to quantitative MRI measures in addition to morphometry metrics used in this study.

### 4.5. Summary

Machine-learning methods are quickly firming themselves as automated diagnostic tools in medicine due to their highly accurate predictions. We have reported an autoencoder-based neural network model that can predict cognitive and clinical status of de novo PD patients over a period of 1 to 4 years. The applicability of the presented model in clinical diagnosis and prognosis is clear and immediate. Our approach relies on well-established neuroimaging software pipelines and is based on low-dimensional features derived from regional DBM. Consequently, the training and testing of our model is far quicker and computationally inexpensive compared to full-stack neural networks that ingest entire imaging volumes and perform prediction on voxel-level information. Although neural network-based predictors are increasingly being used in medical imaging, they are so far confined to giving predictions without providing understanding. Therefore, we should avoid using them merely as “black boxes” that yield different scores for accuracy, specificity, and sensitivity, but also try to interpret their results from a basic science viewpoint. In our study, the input features are directly related to different brain regions, and our saliency analysis offer new insights regarding PD progression and symptomatology. More specifically, we show that the relative contributions of these salient regions change over time, suggesting a dynamical reordering of culprits for each functional impairment as the disease progresses.

## Figures and Tables

**Table 1:** MDS-UPDRS-III Spearman’s rank correlation coefficients of only baseline predictors at four time points past baseline.

Year	Predictor	Type	Side	*R* Value	*p* Value
**1**	MDS-UPDRS-III			0.425	<0.001
	Caudate	SBR	R	−0.209	0.044
	Putamen	SBR	R	−0.351	<0.001
	Hoehn and Yahr			0.292	0.005
	Symbol Digit Modalities Score			−0.28	0.007
	MDS-UPDRS Total Score			0.401	<0.001
	MDS-UPDRS-II PQ			0.293	0.005
	Modified Schwab England ADL			−0.205	0.049
	UPSIT Total Score			−0.243	0.019
	Superior temporal gyrus, anterior part	DBM	R	0.204	0.049
	Subthalamic Nucleus	DBM	R	−0.23	0.026
**2**	MDS-UPDRS-III			0.494	<0.001
	Hoehn and Yahr			0.357	<0.001
	HVLT Immediate Recall			−0.314	0.004
	HVLT Delayed Recognition Hits			−0.274	0.012
	Symbol Digit Modalities Score			-0.229	0.036
	MDS-UPDRS Total Score			0.402	<0.001
	MDS-UPDRS-II PQ			0.291	0.007
	Modified Schwab England ADL			−0.333	0.002
	UPSIT Total Score			−0.224	0.041
	Hippocampus	DBM	L	−0.244	0.022
	Amygdala	DBM	R	−0.12	0.025
	Parahippocampal and ambient gyri	DBM	R	−0.218	0.047
	Inferolateral remainder of parietal lobe	DBM	R	0.22	0.045
	Medial orbital gyrus	DBM	R	0.221	0.044
	Superior temporal gyrus, anterior part	DBM	L	−0.221	0.044
**3**	MDS-UPDRS-III			0.38	<0.001
	Caudate	SBR	R	−0.312	0.004
	Putamen	SBR	R	−0.326	0.002
	Alpha-synuclein			−0.237	0.03
	Hoehn and Yahr			0.353	0.001
	QUIP Positive Buying			−0.223	0.041
	QUIP Positive Hobbies			−0.223	0.041
	MDS-UPDRS Total Score			0.42	<0.001
	MDS-UPDRS-I PQ			0.253	0.02
	MDS-UPDRS-II PQ			0.382	<0.001
	Modified Schwab England ADL			−0.314	0.004
	Fusiform (lateral occipitotemporal) gyrus	DBM	R	0.216	0.048
	Pallidum	DBM	L	0.23	0.035
	Superior frontal gyrus	DBM	L	−0.251	0.021
4	Age			0.281	0.014
	MDS-UPDRS-III			0.51	<0.001
	Caudate	SBR	L	−0.293	0.01
	Caudate	SBR	R	−0.334	0.003
	Putamen	SBR	L	−0.227	0.049
	Putamen	SBR	R	−0.282	0.014
	Hoehn and Yahr			0.248	0.031
	HVLT Delayed Recognition Hits			−0.325	0.012
	Benton Judgement of Line Orientation			−0.302	0.008
	QUIP Positive Eating			0.227	0.049
	MDS-UPDRS Total Score			0.524	<0.001
	MDS-UPDRS-I PQ			0.296	0.01
	MDS-UPDRS-II PQ			0.348	0.002
	SCOPA-AUT			0.255	0.026
	Amygdala	DBM	R	−0.249	0.03
	Gyrus cinguli, posterior part	DBM	R	−0.302	0.008
	Caudate nucleus	DBM	L	−0.309	0.007
	Anterior orbital gyrus	DBM	L	−0.257	0.025
	Posterior orbital gyrus	DBM	L	−0.232	0.043
	Subcallosal area	DBM	L	0.25	0.03
	Subthalamic Nucleus	DBM	R	−0.266	0.02

**Table 2:** MoCA Spearman’s rank correlation coefficients of only baseline predictors at four time points past baseline.

Year	Predictor	Type	Side	*R* Value	*p* Value
**1**	Age			0.373	<0.001
	MoCA			−0.402	<0.001
	Amyloid Beta (1–42)			−0.212	0.034
	HVLT Immediate Recall			−0.422	<0.001
	HVLT Delayed Recognition Hits			−0.252	0.011
	Benton Judgement of Line Orientation			−0.243	0.015
	Semantic Fluency Score			−0.436	<0.001
	Symbol Digit Modalities Score			−0.459	<0.001
	MDS-UPDRS-I			0.214	0.032
	Hippocampus	DBM	R	−0.212	0.034
	Amygdala	DBM	L	−0.209	0.037
	Putamen	DBM	R	−0.221	0.027
	Precentral gyrus	DBM	R	0.289	0.004
	Subthalamic Nucleus	DBM	L	−0.235	0.019
	Subthalamic Nucleus	DBM	R	−0.228	0.022
**2**	Age			0.29	0.003
	MoCA			−0.368	<0.001
	Amyloid Beta (1–42)			−0.269	0.007
	HVLT Immediate Recall			−0.379	<0.001
	HVLT Delayed Recognition Hits			−0.229	0.022
	HVLT Delayed Recognition False Alarms			0.306	0.002
	Semantic Fluency Score			−0.296	0.003
	Symbol Digit Modalities Score			−0.375	<0.001
	MDS-UPDRS Total			0.217	0.029
	MDS-UPDRS-I			0.259	0.009
	MDS-UPDRS-II PQ			0.245	0.014
	Modified Schwab England ADL			−0.26	0.009
	Hippocampus	DBM	R	−0.2	0.045
	Amygdala	DBM	R	−0.215	0.031
	Amygdala	DBM	L	−0.205	0.039
	Putamen	DBM	L	−0.282	0.004
	Putamen	DBM	R	−0.285	0.004
	Thalamus	DBM	R	−0.225	0.024
	Precentral gyrus	DBM	L	−0.21	0.035
	Superior temporal gyrus, anterior part	DBM	R	−0.198	0.047
	Red Nucleus	DBM	L	−0.203	0.042
	Substantia Nigra	DBM	L	−0.236	0.017
	Substantia Nigra	DBM	R	−0.255	0.01
	Subthalamic Nucleus	DBM	L	−0.208	0.037
	Subthalamic Nucleus	DBM	R	−0.28	0.005
**3**	Age			0.44	<0.001
	MDS-UPDRS-III			0.286	0.003
	MoCA			−0.442	<0.001
	SBR Right Putamen			−0.205	0.038
	Hoehn and Yahr			0.206	0.037
	HVLT Immediate Recall			−0.471	<0.001
	HVLT Delayed Recognition Hits			−0.41	<0.001
	Benton Judgement of Line Orientation			−0.235	0.017
	Semantic Fluency Score			−0.51	<0.001
	Symbol Digit Modalities Score			−0.518	<0.001
	MDS-UPDRS Total			0.255	0.009
	UPSIT Total Score			−0.243	0.013
	Hippocampus	DBM	R	−0.197	0.046
	Hippocampus	DBM	L	−0.248	0.012
	Amygdala	DBM	L	−0.24	0.015
	Anterior temporal lobe	DBM	L	−0.217	0.028
	Putamen	DBM	L	−0.324	<0.001
	Putamen	DBM	R	−0.307	0.002
	Thalamus	DBM	L	−0.223	0.023
	Thalamus	DBM	R	−0.272	0.006
	Pallidum	DBM	R	−0.269	0.006
	Red Nucleus	DBM	L	−0.304	0.002
	Red Nucleus	DBM	R	−0.231	0.019
	Substantia Nigra	DBM	L	−0.258	0.009
	Substantia Nigra	DBM	R	−0.252	0.01
	Subthalamic Nucleus	DBM	L	−0.254	0.01
	Subthalamic Nucleus	DBM	R	−0.276	0.005
**4**	Age			0.452	<0.001
	MoCA			−0.438	<0.001
	HVLT Immediate Recall			−0.505	<0.001
	HVLT Delayed Recognition Hits			−0.344	<0.001
	HVLT Delayed Recognition False Alarms			0.219	0.033
	Semantic Fluency Score			−0.424	<0.001
	Symbol Digit Modalities Score			−0.492	<0.001
	MDS-UPDRS Total			0.276	0.007
	MDS-UPDRS-I PQ			0.305	0.003
	UPSIT Total Score			−0.222	0.03
	SCOPA-AUT			0.367	<0.001
	Nucleus accumbens	DBM	R	−0.26	0.012
	Putamen	DBM	L	−0.35	<0.001
	Putamen	DBM	R	−0.322	0.002
	Thalamus	DBM	L	−0.227	0.027
	Thalamus	DBM	R	−0.27	0.009
	Pallidum	DBM	L	−0.207	0.044
	Pallidum	DBM	R	−0.24	0.019
	Red Nucleus	DBM	L	−0.219	0.033
	Red Nucleus	DBM	R	−0.213	0.038
	Substantia Nigra	DBM	L	−0.202	0.049
	Subthalamic Nucleus	DBM	L	−0.252	0.014

## Code Availability

All codes and datasets used in this work are available at https://github.com/Raj-Lab-UCSF/Parkinsonian_Symptomatology_Uncovered.

## Abbreviations

DBM	Deformation-based Morphometry
MCI	Mild Cognitive Impairment
MDS-UPDRS-III	Movement Disorder Society-Unified Parkinson’s Disease Rating Scale Part III
MoCA	Montreal Cognitive Assessment
PD	Parkinson’s Disease
PPMI	Parkinson’s Progression Markers Initiative
